# Cetacean Morbillivirus in Coastal Indo-Pacific Bottlenose Dolphins, Western Australia

**DOI:** 10.3201/eid2004.131714

**Published:** 2014-04

**Authors:** Nahiid Stephens, Pádraig J. Duignan, Jianning Wang, John Bingham, Hugh Finn, Lars Bejder, Anthony P. Patterson, Carly Holyoake

**Affiliations:** Murdoch University, Perth, Western Australia, Australia (N. Stephens, H. Finn, L. Bejder, C. Holyoake);; University of Calgary, Calgary, Alberta, Canada (P.J. Duignan); CSIRO, East Geelong, Victoria, Australia (J. Wang, J. Bingham);; Agri-Food and Biosciences Institute, Belfast, Northern Ireland, UK (I.A.P. Patterson)

**Keywords:** Australia, cetacean, common bottlenose dolphin, dolphin, epizootic, gene amplification, Indian Ocean, Indo-Pacific bottlenose dolphin, immunohistochemical testing, IHC, morbillivirus, Paramyxoviridae, pathology, phylogeny, PCR, Tursiops aduncus, Tursiops truncatus, Western Australia, viruses

## Abstract

Cetacean morbillivirus (CeMV) has caused several epizootics in multiple species of cetaceans globally and is an emerging disease among cetaceans in Australia. We detected CeMV in 2 stranded coastal Indo-Pacific bottlenose dolphins (*Tursiops aduncus*) in Western Australia. Preliminary phylogenetic data suggest that this virus variant is divergent from known strains.

Cetacean morbillivirus (CeMV; family *Paramyxoviridae*) has caused several epizootics globally during the past 25 years. Three strains of CeMV—porpoise, dolphin, and pilot whale morbillivirus—are classified as 1 species (*1*). CeMV is more closely related to ruminant morbilliviruses and human measles virus than to canine and phocine distemper viruses (*1*,*2*).

Recently, a morbillivirus with phylogenetic similarity to dolphin morbillivirus (DMV) caused the death of a common bottlenose dolphin (*Tursiops truncatus*) in Queensland, northeastern Australia (*1*). Retrospective serologic testing from eastern Australia has confirmed seropositivity to morbillivirus in several species; these temporal data suggest circulation of a morbillivirus among cetaceans in this region as long ago as 1985 (*3*). However, whether DMV or variant viruses are circulating in the southwestern Pacific is unknown (*4*). The Queensland reports corroborate studies from 1997 that demonstrated high DMV seroprevalence in long-finned pilot whales (*Globicephala melas*) from northern New Zealand and seropositivity in 1 *T. truncatus* dolphin from Tasmania, Australia (*2*). Seroprevalence of morbillivirus among *G. melas* whales and melon-headed whales (*Peponocephala electra*) from the Tasman Sea (*2*,*3*) is similar to that found among North Atlantic pilot whales, a species in which infection is thought to be endemic (*5*).

Little is known about the prevalence and pathogenicity of CeMV in the Indian Ocean. A low DMV antibody titer by indirect ELISA was reported from a common dolphin (*Delphinus delphis*) from eastern South Africa (CeMV-related pathology was not seen), but no comparable data from Western Australia exist (*2*). We report the deaths of 2 Indo-Pacific bottlenose dolphins (*T. aduncus*) in Western Australia from opportunistic infections secondary to chronic morbillivirus-induced immunosuppression.

## The Study

During June 2009 (midwinter), 3 deaths occurred among a population of ≈20–25 *T. aduncus* dolphins in the Swan River in Western Australia (32°04′S, 115°48′E) (*6*). By comparison, only 6 deaths were recorded during 2002–2008 (D. Coughran, pers. comm.; *7*). Necropsies and histopathologic, bacteriologic, and mycologic testing were performed on 2 of the dolphins (Table); the remains of dolphin 1 were too decomposed for detailed examination. Age class was estimated from morphometrics and reproductive development, supported by dental analysis. The API 20E test kit (bioMérieux, Inc., Boston, MA, USA) was used to identify isolated bacteria.

**Table:** Summary of findings for 3 bottlenose dolphins recovered from the Swan River, Western Australia, Australia*

Dolphin no.	Date recovered	Description	Pathologic findings	IHC results	RT-PCR results
1	2009 Jun 5	Juvenile male in good body condition; TL 210 cm	Too decomposed for necropsy	ND	ND
2	2009 Jun 8	Juvenile male in good body condition; TL 210 cm; postmortem interval ≈72 h	1. Encephalitis: severe, focally extensive, suppurative, and necrotizing, with multifocal vasculitis and thrombus formation, with intra-lesional fungal hyphae†	Widespread positive staining of lymphocytes, mesenteric lymph node‡	Positive for morbillivirus N and P genes§
			2. Splenic and mesenteric node lymphoid depletion, severe		
			3. Segmental jejunal submucosal hemorrhage with thrombosis		
			4. Pulmonary (*Halocercus* sp.) and gastric (*Anisakis* sp.) nematodes; trematode (*Campanula* sp.) infestation of the biliary and pancreatic ducts		
3	2009 Jun 21	Subadult female in good body condition; TL 222.5 cm; postmortem interval ≈72 h	1. Bronchointerstitial pneumonia: multifocal, pyogranulomatous, associated with intralesional fungal hyphae¶	Widespread staining of lymphocytes, mesenteric lymph nodes; weak staining of vascular endothelium, mesenteric lymph node; widespread staining in hepatic Kupffer cells, sinusoidal endothelial cells, and biliary epithelium#	Positive for morbillivirus N and P genes**
			2. Nephritis: multifocal, necrotizing, severe, acute, with intralesional bacteria		
			3. Severe systemic lymphoid depletion		
			4. Fishing line entanglement, right fluke: chronic and proliferative, with granulation tissue formation, dermatitis, and hyperkeratosis		
			5. Pulmonary (*Halocercus* sp.) and gastric (*Anisakis* sp.) nematodes; trematode (*Campanula* sp.) infestation of the biliary and pancreatic ducts		

**Figure 1:**
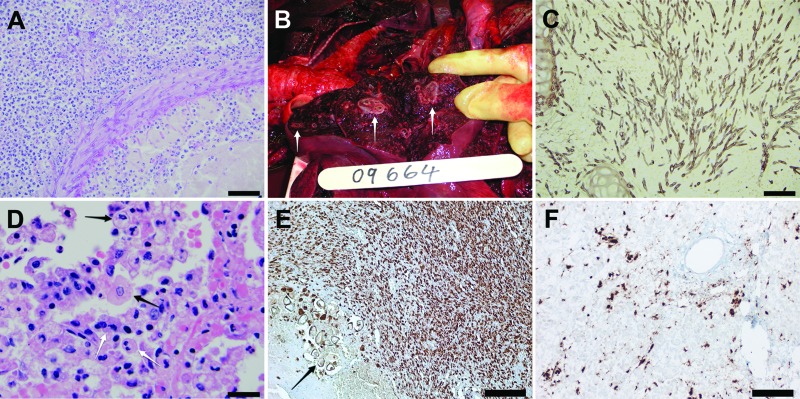
Images of tissue samples from 2 stranded coastal Indo-Pacific bottlenose dolphins (*Tursiops aduncus*) from Western Australia, Australia. A) Brain of dolphin 2 showing cerebral hemisphere with focally extensive suppurative and necrotizing encephalitis surrounding an arteriole. There are intramural and perivascular septate branching hyphae. Hematoxylin and eosin stain. Scale bar = 50 μm. B) Lung of dolphin 3 showing a transected lobar surface exhibiting multifocal pyogranulomas (white arrows). C) Lung of dolphin 3 showing bronchointerstitial pneumonia with branching septate hyphae within a bronchiolar lumen and surrounding the bronchiolar cartilage. Grocott hexamine silver. Scale bar = 50 μm. D) Lung of dolphin 3 showing alveolar lumens filled with desquamated pneumocytes, macrophages, and neutrophils. Enlarged macrophages are occasionally binucleate (white arrows) and rarely exhibit eosinophilic intracytoplasmic inclusions or margination of chromatin and eosinophilic intranuclear inclusions (black arrows). Hematoxylin and eosin stain. Scale bar = 20 μm. E) Mesenteric lymph node of dolphin 2 showing intense staining of morbilliviral antigen in lymphocytes within the cortex. Thick-walled structures (arrow) are trematode eggs. DAB and hematoxylin stain. Scale bar = 200 μm. F) Liver of dolphin 3 showing morbillivirus antigen in Kupffer cells and sinusoidal endothelial cells. DAB and hematoxylin stain. Scale bar = 100 μm.

**Figure 2:**
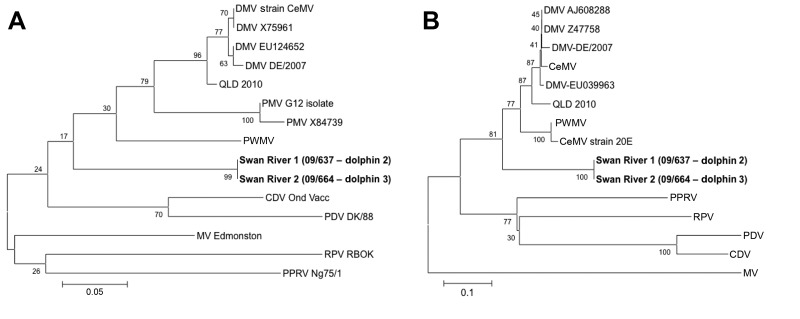
Phylogenetic trees showing partial sequences of morbillivirus nucleoprotein (A) and phosphoprotein (B) genes of cetacean morbillivirus (CeMV) isolates found in 2 stranded coastal Indo-Pacific bottlenose dolphins (*Tursiops aduncus*) from Western Australia, Australia (boldface), and those of other known morbilliviruses. Trees were generated by the neighbor-joining method; bootstrap (1,000 replicates) values of >50 are indicated at the internal nodes. The length of each pair of branches represents the distance between the sequence pairs. Scale bars indicate percentage of nucleotide differences. DMV, dolphin morbillivirus; QLD, Queensland (Australia); PMV, porpoise morbillivirus; PWMV, pilot whale morbillivirus; CDV, canine distemper virus; OND vacc, Onderstepoort strain (used for vaccination); PDV, phocine distemper virus; MV, measles virus; RPV, rinderpest virus; PPRV, peste des petits ruminants virus.

*Located at 32°04′S, 115°48′E. TL, total length in centimeters; IHC, immunohistochemical; RT-PCR, reverse transcription PCR; ND, not done; N, nucleoprotein; P, phosphoprotein. †See Figure 1, panel A. ‡See Figure 1, panel E. §See Figure 2, Swan River 1. ¶See Figure 1, panels B–D. #See Figure 1, panel F. **See Figure 2, Swan River 2.

Both dolphins showed systemic lymphoid depletion. The cause of death for dolphin 2, a juvenile male (Table), was severe, focally extensive cerebral necrosis, secondary to vasculitis and thrombosis associated with abundant fungal hyphae characteristic of *Aspergillus* spp. (Figure 1, panel A); however, we could not culture the organism collected. Dolphin 3, a subadult female, died as a result of multifocal pyogranulomatous bronchopneumonia affecting ≈30% of the lungs; intralesional hyphae characteristic of *Aspergillus* spp. were found (Figure 1, panels B and C). At the periphery of the mycotic lesions were foci of type II pneumocyte hyperplasia, septal fibroplasia, and mononuclear infiltration. Occasional macrophages exhibited chromatin margination and intranuclear eosinophilic inclusions; syncytia were not seen (Figure 1, panel D). Multifocal bilateral renal necrosis associated with bacteria affected ≈30%–40% of the parenchyma. Using the API 20E test kit, we isolated *Staphylococcus aureus* and a gram-negative bacillus similar to *Mannheimia haemolytica* (67%) or *Morganella morganii* (26%). *Penicillium* spp., a presumptive contaminant, was the only fungus isolated.

Immunohistochemical (IHC) testing was conducted by using a monoclonal antibody for canine distemper virus nucleoprotein (VMRD, Inc., Pullman, WA, USA) diluted at 1:100, according to published protocols (*8*). Specific positive staining was found in multiple tissues from dolphins 2 and 3, including lymphocytes within lymph nodes, hepatic sinusoidal endothelial cells and Kupffer cells, biliary epithelium, and tunica media myocytes of blood vessels within the liver and mesenteric lymph nodes (Figure 1, panels E and F).

Reverse transcription PCR (RT-PCR) was performed as described (*1*). Amplified products (≈238 bp from the nucleoprotein [N] gene and ≈425 bp from the phosphoprotein [P] gene) were sequenced and compared with sequences of DMV and other morbilliviruses by using BLASTN (http://blast.ncbi.nlm.nih.gov/Blast.cgi). Identical sequences of the highly conserved N and P genes were obtained from dolphins 2 and 3; these genes showed 79%–83% (dolphin 2) and 75%–79% (dolphin 3) nucleotide identity to sequences of CeMV strains from GenBank. Nucleotide comparison between the N and P gene sequences from the dolphins from Western Australia and the dolphin from Queensland (*1*) revealed only 83% and 79% similarity, respectively. Partial N and P gene sequencing and analysis using MEGA5 software (*1*) indicated that the Western Australia variant differed from other cetacean morbilliviruses and represents a distinct lineage (Figure 2).

## Conclusions

We found CeMV in dolphins that died in the Indian Ocean in 2009; this finding thus predates reports of the virus in animals on the eastern coast of Australia (*1*,*3*) and recent confirmation of the virus in South Australia (C. Kemper and I. Tomo, pers. comm.). The virus we found is phylogenetically distinct from that isolated from cetaceans in eastern Australia. Complete sequencing of viruses from all Australian regions is needed, but preliminary data suggest that the variant from Queensland clusters with viruses isolated from cetaceans in the Northern Hemisphere, whereas the variant from Western Australia is distinct from other morbilliviruses—closely related to, but divergent from, other cetacean morbilliviruses. Our data also suggest that the variant from Western Australia is the least divergent of all cetacean morbilliviruses from the terrestrial viruses rinderpest, peste des petit ruminants, and measles. Rinderpest is considered the archetypal genus member (*9*); our data suggest that the Western Australia variant is the most closely related cetacean morbillivirus to the terrestrial members of the genus.

Lesions in the dolphins we examined were not those typically seen in classic acute morbillivirus infections, and diagnosis required IHC testing and RT-PCR. The signs we saw may be more common for infections that occur in temperate/tropical waters, wherein infected animals survive the viral infection but succumb to opportunistic infections facilitated by virus-induced chronic immunosuppression following lymphoid depletion. During mass mortality events involving various species that were ultimately attributed to CeMV on the US Atlantic coast (1982, 1987–1988) and Gulf Coast (1993–1994) (*10*,*13*) and in the Mediterranean Sea (1990–1992 and 2006–2007) (*10*), opportunistic infections were common (*1*,*10*). In 1 study, antigen was detectable by IHC testing in just 53% of cases (*11*); RT-PCR increased detection rates to >90% in autolysed tissues or for cases in which the acute phase had passed and few to no pathognomonic lesions remained (*11*).

Diagnosis of CeMV is a challenge in areas in which epidemics have not been recorded, in CeMV-endemic areas in which clinical cases are rare, or in new hosts. Subclinical infection may play a more critical epidemiologic role than previously thought, further complicating assessment of deaths (*12*). Archived serum samples from the western Atlantic documented a pattern of recurrent mortality events in bottlenose dolphins dating back to 1982 (*13*); investigations have also shown that CeMV may persist between outbreaks without causing clinical disease (*12*). Infection may thus become endemic, and periodic incursions into immunologically naive populations may cause deaths at epidemic rates (*12*). A similar scenario of virus circulation without clinical disease has been proposed for common dolphins (*D. delphis*) in the eastern North Pacific (*14*) and for Mediterranean striped dolphins (*Stenella coeruleoalba*) and pilot whales (*G. melas*) (*10*,*15*). A 30-year study of strandings in Western Australia (1981–2010) showed a pattern of mortality peaks and troughs in bottlenose dolphins similar to that seen along the US Atlantic coast (*7*,*13*). A peak in 2009 coincided with the cases we report. Further study of CeMV circulation and spread is needed.

## References

[1] Stone BM, Blyde DJ, Saliki JT, Blas-Machado U, Bingham J, Hyatt A, Fatal cetacean morbillivirus infection in an Australian offshore bottlenose dolphin (*Tursiops truncatus*). Aust Vet J. 2011;89:452–7 . 10.1111/j.1751-0813.2011.00849.x 22008125

[2] Van Bressem MF, Van Waerebeek K, Jepson PD, Raga JA, Duignan PJ, Neilsen O, An insight into the epidemiology of dolphin morbillivirus worldwide. Vet Microbiol. 2001;81:287–304. 10.1016/S0378-1135(01)00368-6 11390111

[3] Stone BM, Blyde DJ, Saliki JT, Morton JM. Morbillivirus infection in live stranded, injured, trapped and captive cetaceans in southeastern Queensland and northern New South Wales, Australia. J Wildl Dis. 2012;48:47–55. 10.7589/0090-3558-48.1.47 22247373

[4] West KL, Sanchez S, Rotstein D, Robertson KM, Dennison S, Levine G, A Longman’s beaked whale (*Indopacetus pacificus*) strands in Maui, Hawaii, with first case of morbillivirus in the central Pacific. Mar Mamm Sci. 2012;29:767–76.

[5] van Bressem MF, Jepson PD, Barrett T. Further insight on the epidemiology of cetacean morbillivirus in the northeastern Atlantic. Mar Mamm Sci. 1998;14:605–13. 10.1111/j.1748-7692.1998.tb00747.x

[6] Chabanne D, Finn H, Salgado-Kent C, Bejder L. Identification of a resident community of bottlenose dolphins (*Tursiops* spp.) in the Swan-Canning Estuary, Western Australian, using behavioural information. Pac Conserv Biol. 2012;18:247–62.

[7] Groom CJ, Coughran DK. Three decades of cetacean strandings in Western Australia: 1981 to 2010. J R Soc West Aust. 2012;95:63–76.

[8] Kennedy S, Smyth JA, Cush PF, McAliskey M, McCullough SJ, Rima BK. Histopathologic and immunocytochemical studies of distemper in harbor porpoises. Vet Pathol. 1991;28:1–7. 10.1177/030098589102800101 2017822

[9] Pomeroy LW, Bjørnstad ON, Holmes EC. The evolutionary and epidemiologic dynamics of the paramyxoviridae. J Mol Evol. 2008;66:98–106. 10.1007/s00239-007-9040-x 18217182 PMC3334863

[10] van Bressem MF, Raga JA, Di Guardo G, Jepson PD, Duignan PJ, Siebert U, Emerging infectious diseases in cetaceans worldwide and the possible role of environmental stressors. Dis Aquat Organ. 2009;86:143–57. 10.3354/dao02101 19902843

[11] Krafft A, Lichy JH, Lipscomb TP, Klaunberg BA, Kennedy S, Taubenberger JK. Postmortem diagnosis of morbillivirus infection in bottlenose dolphins (*Tursiops truncatus*) in the Atlantic and Gulf of Mexico epizootics by polymerase chain reaction–based assay. J Wildl Dis. 1995;31:410–5. 10.7589/0090-3558-31.3.410 8592367

[12] Bossart GD, Reif JS, Schaefer AM, Goldstein J, Fair PA, Saliki JT. Morbillivirus infection in free-ranging Atlantic bottlenose dolphins (*Tursiops truncatus*) from the southeastern United States: seroepidemiologic and pathologic evidence of subclinical infection. Vet Microbiol. 2010;143:160–6. 10.1016/j.vetmic.2009.11.024 20005646

[13] Duignan PJ, House C, Odell DK, Wells RS, Hansen MT. Morbillivirus in bottlenose dolphins: evidence for recurrent epizootics in the western Atlantic and Gulf of Mexico. Mar Mamm Sci. 1996;12:499–515. 10.1111/j.1748-7692.1996.tb00063.x

[14] Reidarson TH, McBain J, House C, King DP, Stott JL, Krafft A, Morbillivirus infection in stranded common dolphins from the Pacific Ocean. J Wildl Dis. 1998;34:771–6. 10.7589/0090-3558-34.4.771 9813847

[15] Banyard AC, Tiwari A, Barrett T. Morbillivirus infection in pilot whales: Strict protein requirement drives genetic conservation. Arch Virol. 2011;156:1853–9. 10.1007/s00705-011-1042-8 21671040

